# Supplementary material for: Polycomb group ring finger protein 6 suppresses Myc-induced lymphomagenesis
Source: Life Sci Alliance. 2022 Apr 14;5(8):e202101344. doi: 10.26508/lsa.202101344 (PMC9012912; doi:10.26508/lsa.202101344)
Supplement: Supplementary file 1 [file LSA-2021-01344_TableS1.docx]

**Supplemental Tables**

**Table S1A. Breeding: CD19-*Cre/Cre; Pcgf6^+/fl^* x Eμ-*myc; Pcgf6^+/fl^***

| **Genotype** | **Expected frequency** | **Observed frequency** | |
| --- | --- | --- | --- |
| CD19-*Cre; Pcgf6^+/+^* | 12.50% | 21/173 | 12.14% |
| CD19-*Cre; Pcgf6^+/fl^* | 25% | 52/173 | 30.06% |
| CD19-*Cre; Pcgf6^fl/fl^* | 12.50% | 30/173 | 17.34% |
| CD19-*Cre*; Eμ-*myc*; *Pcgf6^+/+^* | 12.50% | 36/173 | 20.81% |
| CD19-*Cre*; Eμ-*myc*; *Pcgf6^+/fl^* | 25% | 29/173 | 16.76% |
| CD19-*Cre*; Eμ-*myc*; *Pcgf6^fl/fl^* | 12.50% | 5/173 | 2.89% |

**Table S1B. Breeding: CD19*-Cre; Mga^+/fl^* x Eμ-*myc; Mga^+/fl^***

| **Genotype** | **Expected frequency** | **Observed frequency** | |
| --- | --- | --- | --- |
| *Mga^+/+^* | 6.25% | 6/96 | 6.25% |
| *Mga^+/fl^* | 12.50% | 23/96 | 23.96% |
| *Mga^fl/fl^* | 6.25% | 2/96 | 2.08% |
| Eμ-*myc*; *Mga^+/+^* | 6.25% | 7/96 | 7.29% |
| Eμ-*myc*; *Mga^+/fl^* | 12.50% | 3/96 | 3.13% |
| Eμ-*myc*; *Mga^fl/fl^* | 6.25% | 9/96 | 9.38% |
| CD19-*Cre*; *Mga^+/+^* | 6.25% | 11/96 | 11.46% |
| CD19-*Cre*; *Mga^+/fl^* | 12.50% | 19/96 | 19.79% |
| CD19-*Cre*; *Mga^fl/fl^* | 6.25% | 3/96 | 3.13% |
| CD19-Cre; Eμ-*myc*; *Mga^+/+^* | 6.25% | 4/96 | 4.17% |
| CD19-Cre; Eμ-*myc*; *Mga^+/fl^* | 12.50% | 5/96 | 5.21% |
| CD19-Cre; Eμ-*myc*; *Mga^fl/fl^* | 6.25% | 4/96 | 4.17% |

**Table S1. Breeding Strategy.** Each table shows the expected (assuming mendelian distribution) and observed frequencies of the indicated compound genotypes, based on the crosses shown at the top. **(A**) *Pcgf6* mutant cohort. Here all siblings are positive for CD19-*Cre*, as this transgene was first bred to homozygosity in one of the parents (CD19-*Cre/Cre*). Note that Eμ-*myc* and *Pcgf6* segregated in a sub-Mendelian manner  (p <0.0001 ), consistent with their close genomic location on chromosome 19 (Lefebure et al. 2017) (<http://www.informatics.jax.org/marker/MGI:1918291>). **(B**) *Mga* mutant cohort. In line with published data (Washkowitz et al. 2015), *Mga^fl/fl^* mice were recovered at sub-Mendelian frequencies (p < 0.005), confirming that *Mga^fl^* is a hypomorphic allele.
