# Supplementary figures and images for: Polycomb group ring finger protein 6 suppresses Myc-induced lymphomagenesis
Source: Life Sci Alliance. 2022 Apr 14;5(8):e202101344. doi: 10.26508/lsa.202101344 (PMC9012912; doi:10.26508/lsa.202101344)

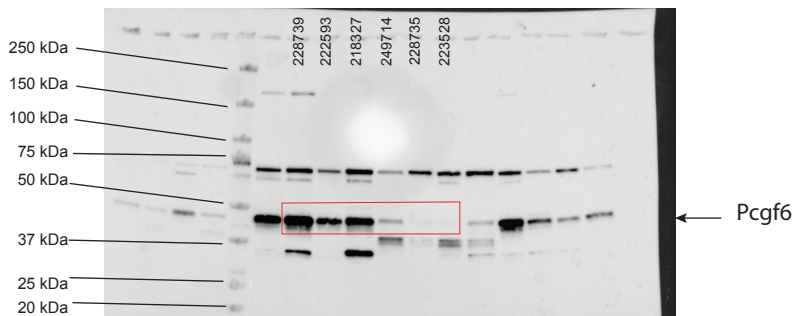

Exposure time 120 sec

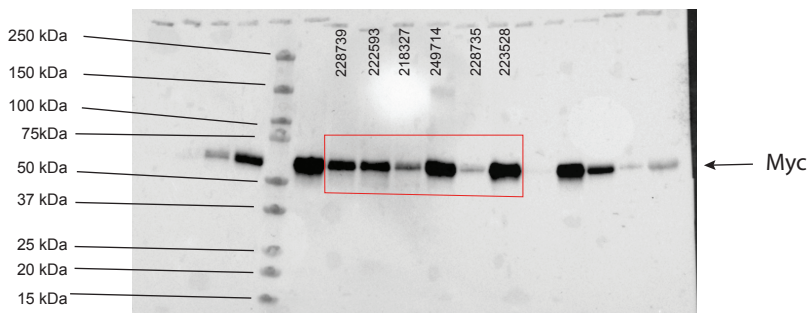

Exposure time 593.8 sec

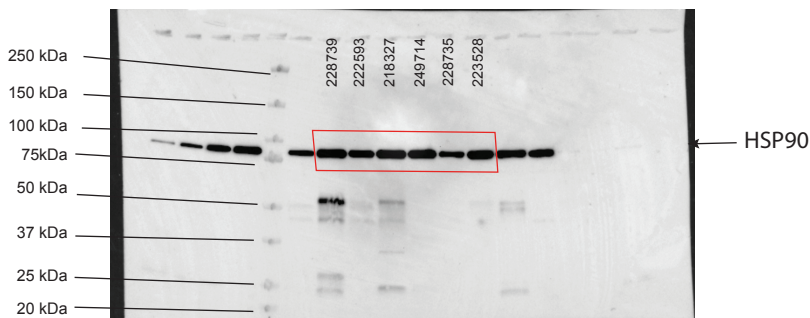

Exposure time 220 sec

Supplement: Supplementary file 2 [file LSA-2021-01344_SdataF1.pdf]
