# Supplementary material for: Polycomb group ring finger protein 6 suppresses Myc-induced lymphomagenesis
Source: Life Sci Alliance. 2022 Apr 14;5(8):e202101344. doi: 10.26508/lsa.202101344 (PMC9012912; doi:10.26508/lsa.202101344)
Supplement: Supplementary file 6 [file LSA-2021-01344_SdataFS4.pdf]

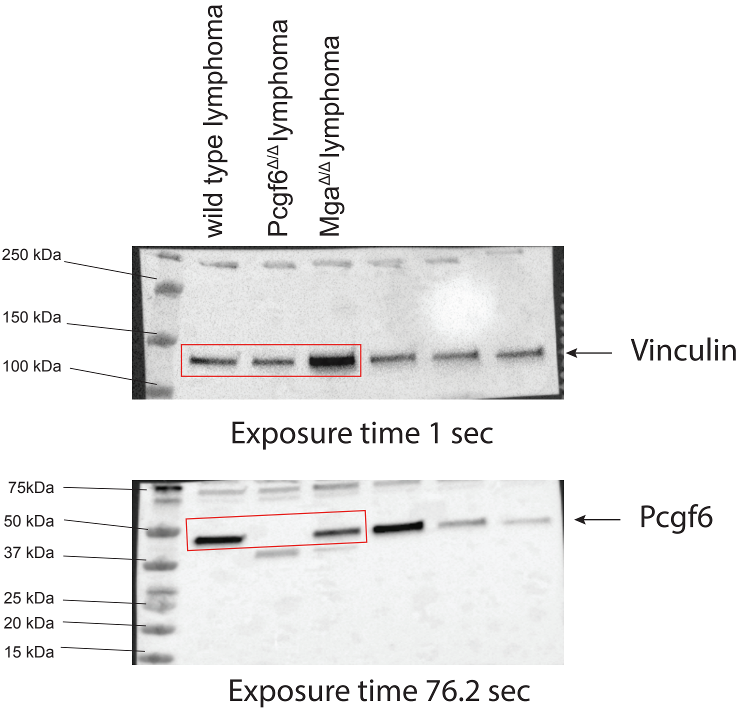
wild type lymphoma
Pcgf6$^{\Delta/\Delta}$ lymphoma
Mga$^{\Delta/\Delta}$ lymphoma
250 kDa
150 kDa
100 kDa
Vinculin
Exposure time 1 sec
75kDa
50 kDa
37 kDa
25 kDa
20 kDa
15 kDa
Pcgf6
Exposure time 76.2 sec
