# Supplementary material for: Polycomb group ring finger protein 6 suppresses Myc-induced lymphomagenesis
Source: Life Sci Alliance. 2022 Apr 14;5(8):e202101344. doi: 10.26508/lsa.202101344 (PMC9012912; doi:10.26508/lsa.202101344)
Supplement: Supplementary file 7 [file LSA-2021-01344_TableS5.docx]

**Table S5A. Antibodies for immunoblotting**

| **Antibody** | **Source** | **Dilution/mg used** |
| --- | --- | --- |
| Myc Y69 | Abcam (ab32072) | 1:2000 |
| Vinculin | Sigma-Aldrich (V9264) | 1:10000 |
| Max | Bethyl (A302-866A) | 1:1000 |
| Pcgf6 | Scelfo et al. 2019 | 1:1000 |
| Hsp90 | Santa Cruz (sc-13119) | 1:10000 |

**Table S5B. Antibodies for flow cytometric analysis**

| **Antibody** | **Conjugate** | **Company** | **Clone** | **Dilution used** |
| --- | --- | --- | --- | --- |
| B220 | PE | BD Pharmingen (#553089) | RA3-6B2 | 1:200 |
| B220 | efluor 450 | eBioScience (48-0452-82) | RA3-6B2 | 1:200 |
| CD19 | PE-Cy7 | BD Pharmigen (552854) | 1D3 | 1:400 |
| CD21 | PE | eBioScience (12-0211-82) | 8D9 | 1:800 |
| CD23 | AlexaFluor 647 | BioLegend (101612) | B3B4 | 1:100 |
| CD25 | APC | eBioScience (17-0251-82) | PC61.5 | 1:100 |
| CD43 | FITC | eBioScience (11-0431-85) | R2/60 | 1:200 |
| IgD | BV510 | BD Pharmigen (#563110) | 11-26C.2A | 1:200 |
| IgM | APC | eBioScience (47-5790-82) | II/41 | 1:200 |
| IgM | APC-Cy7 | BioLegend (406516) | RMM-1 | 1:200 |
| CD45 | BV786 | BD Pharmingen (#564225) | 30-F11 | 1:!00 |

**Table S5.** List of materials used in this work *(continued on the next page).*

**Table S5C. Antibodies for chromatin immunoprecipitation.**

| **Antibody** | **Company** |
| --- | --- |
| Max | Bethyl (A302-866A) |
| Pcgf6 | Scelfo et al. 2019 |
| Myc N262 | Santa Cruz (sc-764) |
| H3K4me3 | Active Motif (#39159) |
| H3K4me1 | Abcam (ab8895) |
| H3K27ac | Abcam (ab4729) |
| H3K27me3 | Cell Signaling (#9733) |
| IgG | Santa Cruz (sc-2027) |
| H2AK119Ub | Cell Signaling |

**Table S5D. PCR Primers.**

| **Application** | **Locus** | **Forward primer** | **Reverse primer** | **Amplicon (bp)** |
| --- | --- | --- | --- | --- |
| **Expression  (RT-PCR)** | mouse *Pcgf6* | CTTCTCTCTGCGTCTGGAGTC | TCAGCTCGACAAGGTTTATCAG | 77 |
|  | mouse *Myc* | TTTTTGTCTATTTGGGGACAGTG | CATCGTCGTGGCTGTCTG | 130 |
|  | mouse *Max* | CCTGGGCCGTAGGAAATGAG | CAGCCGCAGATTGAAACCTC | 82 |
|  | mouse *Mga* | AAATCTTTAACTGCTGCCAAGAA | CTGCAACCTGAATCATTTGTGGT | 200 |
|  | mouse *H3* | GTGAAGAAACCTCATCGTTACAGGCCTGGT | CTGCAAAGCACCAATAGCTGCACTCTGGAA | 177 |
| **Analysis of recombination on gDNA by semi-quantitative PCR** | *Mga^wt^* | ATTCCTGTAGGCCCTGGAAG | GGGAGGATTGGGAAGACAAT | 325 |
|  | Mga*^Δ^* | ATTCCTGTAGGCCCTGGAAG | CAGGACAACCTGACACCTCTG | 600 |
|  | Loading Control (*Il2*) | CTAGGCCACAGAATTGAAAGATCT | GTAGGTGGAAATTCTAGCATCATCC | 324 |
| **Genotyping** | *CD19* wt | CCAGACTAGATACAGACCAG | AACCAGTCAACACCCTTCC | 452 |
|  | *CD19-Cre* | CCAGACTAGATACAGACCAG | TCAGCTACACCAGAGACGG | 750 |
|  | Eµ-*myc* | GGTTTAATGAATTTGAAGTTGCCA | TTCTTGCCCTGCGTATATCAGTC | 210 |
|  | *Mga^wt^* | CAGGACAACCTGACACCTCTG | GGTATGGTTGTAATGATCAGCTTTC | 325 |
|  | *Mga^fl^ (Mga^inv^)* | CAGGACAACCTGACACCTCTG | GCTGGGGCTCGATCCTCTAG | 500 |
|  | *Pcgf6^wt^* | TTAATTGCTGCGTTCCATCTC | ATGTCAGAGAACTGGGACCGC | 404 |
|  | *Pcgf6^fl^* | TTAATTGCTGCGTTCCATCTC | GGCTAGATCTGCTGGAGACTT | 378 |

**Table S5.** List of materials used in this work.
